# Emotion Recognition Algorithm Application Financial Development and Economic Growth Status and Development Trend

**DOI:** 10.3389/fpsyg.2022.856409

**Published:** 2022-02-28

**Authors:** Dahai Wang, Bing Li, Xuebo Yan

**Affiliations:** ^1^College of Management, Ocean University of China, Qingdao, China; ^2^School of Software, Jiangxi Normal University, Nanchang, China; ^3^College of Artificial Intelligence, East China University of Technology, Nanchang, China

**Keywords:** emotion recognition algorithm, financial development, economic growth, development forecast, application

## Abstract

Financial market and economic growth and development trends can be regarded as an extremely complex system, and the in-depth study and prediction of this complex system has always been the focus of attention of economists and other scholars. Emotion recognition algorithm is a pattern recognition technology that integrates a number of emerging science and technology, and has good non-linear system fitting capabilities. However, using emotion recognition algorithm models to analyze and predict financial market and economic growth and development trends can yield more accurate prediction results. This article first gives a detailed introduction to the existing financial development and economic growth status and development trend forecasting problems, and then gives a brief overview of the concept of emotion recognition algorithms. Then, it describes the emotion recognition methods, including statistical emotion recognition methods, mixed emotion recognition methods, and emotion recognition methods based on knowledge technology, and conducts in-depth research on the three algorithm models of statistical emotion recognition methods, they are the support vector machine algorithm model, the artificial neural network algorithm model, and the long and short-term memory network algorithm model. Finally, these three algorithm models are applied to the financial market and economic growth and development trend prediction experiments. Experimental results show that the average absolute error of the three algorithms is below 25, which verifies that the emotion recognition algorithm has good operability and feasibility for the prediction of financial market and economic growth and development trends.

## Introduction

Financial development is the core of economic growth, and its development trend has always been the focus of the government and countless market investors. The sound development of the financial market and economy can promote the sustainable development of society and people’s lives. From the perspective of contemporary market and economic relations, the good or bad of the financial market’s development momentum determines the quality of economic development to a certain extent. The *status quo* and development trend of financial development and economic growth have not only gradually become an important wind indicator for predicting the healthy development of society, but also represent the people’s expectations and confidence in economic life. On the contrary, the sluggish and sluggish financial market means that the people will face more arduous challenges in the future economic life. Predicting the current status and development trend of financial development and economic growth has become a real demand. However, in actual operation, it is a very difficult task to evaluate the current development and predict the development trend. The current financial and economic development and future development trends are affected by the complexity of the market, and there are many factors that interfere with judgments, and their direction is elusive. How to accurately and quickly predict future economic development and financial market trends, scientifically analyze and study the essential laws of financial development and economic growth, and maintain the sustained and healthy development and operation of financial markets and the economy has become an urgent task.

Emotion recognition algorithm is the product of the current combination of computer technology and artificial intelligence technology. With the continuous maturity and improvement of technology, emotion recognition technology has also been further developed. At present, it is integrated with multiple fields, it is widely used in medical and health, criminal investigation, video tracking and other fields and has achieved remarkable application results. There are many kinds of emotion recognition algorithms, the most important ones are K-means clustering algorithm, SVM recognition algorithm, regression analysis method and so on. These advanced algorithms can realize fast data collection and processing, and can accurately output analysis results. They are extremely versatile, and applying them to the study of financial development and economic growth status and development trends can effectively solve the problem of complex factors interfering with prediction.

This paper proposes a novel research direction of financial development and economic growth status and development trend based on emotion recognition algorithm, and provides economists with a data analysis platform based on intelligent science and technology. This platform can effectively make accurate judgments on the financial market and economic development, and can also provide new ideas for research in the financial and economic fields.

## Related Work

In recent years, many scholars have carried out research on emotion recognition algorithms. Zhao Z proposed an emotion recognition algorithm based on deep learning methods, which combines knowledge transfer and self-attention of SER tasks. He applied the algorithm to deltas and delta-deltas as the input log-Mel spectrogram. In addition, given that emotions are time-dependent, he uses Temporal Convolutional Neural Networks (TCN) to simulate changes in emotions, and further introduces a self-attention algorithm-based attention shift mechanism to study long-term dependence. In the method he proposed, the self-attention transfer network (SATN) uses the attention autoencoder to track attention from the source task; then learns attention from speech recognition, and then transfers this knowledge to SER; finally he passes the evaluation experiment of Interactive Emotional Binary Motion Capture (IEMOCAP) has proved the effectiveness of the new model (Zhang et al., 2021). Li Y proposed AI-EmoCom. He used emotion as a communication medium in the network, combined with artificial intelligence technology to make the emotional communication system more intelligent, and applied the emotional communication system based on artificial intelligence to the field of unmanned driving. He proposed that people-oriented hybrid driving reduces the incidence of traffic accidents to a greater extent. He also applies this algorithm to emotional social robots to provide users with personalized service emotions. Then he introduced in detail the system architecture based on artificial intelligence emotional communication, elaborated on the label-free learning model of data set annotation and processing and the artificial intelligence algorithm model of emotion recognition. And through experiments to verify the interaction delay of the algorithm system and the accuracy of emotion recognition (Li et al., 2019). Fang W C proposed a real-time emotion recognition hardware system architecture for electroencephalogram (EEG) based on multiphase convolutional neural network (CNN) algorithm. The algorithm is implemented on a 28-nanometer technology chip and a field programmable gate array (FPGA) for binary and quaternary classification, and sample entropy, differential asymmetry, short-time Fourier transform and channel reconstruction methods are used for emotional feature extraction. In this work, he selected six EEG channels (FP1, FP2, F3, F4, F7, and F8) and fused the spectrogram to generate EEG images, finally, the effectiveness of the emotion recognition algorithm system is verified through experiments (Fang et al., 2019). Qian Y proposed a new AIEM (AI-enabled emotional experience management) method, he integrated AI and CEM in emotional recognition and interactive intelligent applications. The article introduces the composition and architecture of AIEM from three aspects: intelligent management of emotional data collection, accuracy management of emotion recognition, and real-time management of emotional interaction. Then he used advanced algorithms and models in the two stages of emotion recognition algorithm and emotion computing offloading, and, respectively, chose two deep learning algorithms (VGG-Net and Alex-Net) for facial expression recognition and voice emotion recognition. The final experimental results show that his method can provide intuitive and reasonable user experience management, and select the appropriate computing node for the user (Qian et al., 2018). Agarwal G proposed a speech emotion recognition technology based on optimized deep neural network. He proposed a novel adaptive wavelet transform by using an improved galactic swarm optimization algorithm (AWT_MGSO). It denoises the speech signal and compares it with classifiers such as DNN_DHO, DNN (deep neural network), DAE (deep autoencoder), etc. The final experimental results show that the maximum accuracy of the algorithm is 97.85% on the TESS data set; 97.14% on the RAVDESS data set; 93.75% on the IITKGP-SEHSC data set through the DNN-HHO classifier, which has good feasibility (Agarwal and Om, 2021). Wei P proposed a speech emotion recognition algorithm based on improved stacked kernel sparse depth model, which uses autoencoder, denoising autoencoder and sparse autoencoder to improve Chinese speech emotion recognition. The first layer of the algorithm structure uses the denoising autoencoder learning ratio to input hidden features with larger feature dimensions, and the second layer uses the sparse autoencoder to learn sparse features. Finally, the wavelet kernel sparse SVM classifier is applied to classify the features, and then evaluated on the test data set, which contains spontaneous, non-prototypical and long-term speech emotion data. The final experimental results show that the proposed algorithm has advantages in speech emotion recognition (Wei and Zhao, 2019). In summary, after recent years of exploration, emotion recognition algorithms have been deeply studied by many scholars, but there are not many studies on the *status quo* and development trends of financial development and economic growth. Therefore, in order to further promote the development of society, the practical research on emotion recognition algorithms and the *status quo* of financial development and economic growth brooks no delay.

## Financial Development and Economic Growth Status and Development Trend Forecasting Problems and Emotion Recognition Algorithms

### Financial Development and Economic Growth Status and Development Trend Forecasting Problems

The stock market is a dynamic and non-linear system disturbed by various factors. There are many problems in stock forecasting that have yet to be solved, mainly in the following aspects:

**(1) There is a lot of noise in stock variables**

The financial market dynamic system is huge and complex. Stock prices are not only affected by internal market factors, but also restricted by external factors. For example, the time series of financial market development usually contains a lot of noise (Wu et al., 2021). And there are many techniques used to predict the direction of stocks in the market. In such a complex environment, the more forecasting techniques used for certain financial products or designated stocks are selected. The more noise there is, the more susceptible the prediction process is to interference and influence. These disturbances and influences not only limit the speed of stock price forecasts, but also limit the accuracy of stock price forecasts. Therefore, how to correctly select predictor variables and how to preprocess the noise in the time series is one of the most important prediction problems at present.

**(2) The stock price is obviously non-linear**

The trend of the financial market has non-linear characteristics, especially reflected in the rise and fall of stock prices and the relationship between various internal and external factors that affect stock prices. It can be seen from this that if a technology wants to accurately predict the financial market and economic growth and development trend, it must have strong non-linear processing capabilities (Laskar and Laskar, 2019). But the real problem is that there are not many forecasting techniques that can deal with non-linear problems. Many traditional forecasting techniques can only deal with general linear problems, and it is obvious that accurate results cannot be obtained when they are used in financial market forecasts.

**(3) Stock investors have subjective initiative**

The direction of the market is not only affected by the government’s macro-control and the development of small and medium-sized enterprises, but also by the subjective initiative of investors. Their decisions are often accompanied by strong subjective wishes, and investors’ investment decisions and psychological expectations on the market also affect their predictions on the financial market to a certain extent. Therefore, in the process of forecasting, it is necessary to consider not only objective factors, but also these subjective factors to find out the objective laws of market operation and development (Wang, 2019).

**(4) Uncertainty of prediction**

One of the reasons why financial development and economic growth status and development trend forecasts are so difficult to achieve is also the uncertainty of economic market development. Therefore, choosing appropriate methods and technologies to analyze and predict it has always been the focus of attention of many economists (Yerigeri and Ragha, 2021). Through the information data presented by the market, the algorithm model is simulated and fitted, and then the information outside the data is predicted, to measure whether an algorithm model has the strength and whether the prediction error meets the standard requires an appropriate evaluation standard. However, in the actual prediction process, the algorithm model may not be able to perform a perfect simulation fit to the information data. Therefore, the construction of the profit and loss function is not the main problem. The most important problem to be solved is how to construct a suitable algorithm model to achieve more accurate forecast output.

**(5) The particularity of the stock market**

The development of the financial market began in the second half of the last century, and it took more than two decades to formally take shape until the end. Compared with many Western European and American countries, such development is relatively backward. Coupled with the special structure, the entire market is irregular and non-cyclical, and the internal complex problems (personal investment occupies a high position, investors have strong differences in decision-making, etc.) make market forecasts even more difficult. How to overcome these factors and successfully realize the prediction of the market is also an urgent problem to be solved (Kopaczka et al., 2019).

### Overview of Emotion Rrecognition Algorithms

**FIGURE 1:**
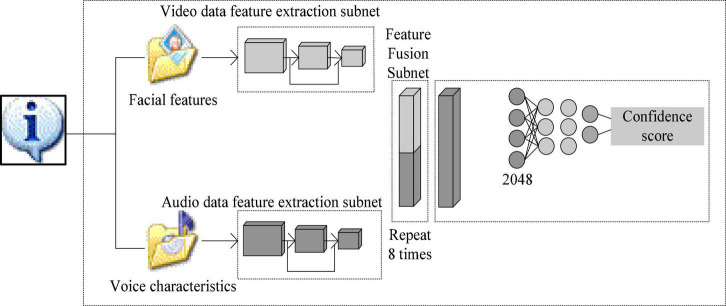
Emotion recognition algorithm structure picture.

Emotion recognition algorithm is a pattern recognition technology developed based on information technology. The execution process of the algorithm is shown in Figure 1. It is mainly to first collect the information data in the database, and then establish an appropriate model to extract the collected data feature information. Finally, a classifier is constructed based on the extracted feature information to obtain the result of emotion recognition. The execution process of the algorithm combines a number of information technologies including computer control technology, signal processing technology, deep learning recognition technology, etc. (Gomathy, 2021).

### Emotion Recognition Algorithm Classification

At present, with the in-depth development of emotion recognition, more and more research methods and technologies are trying to combine with emotion recognition, for example, mutual information method, Bayesian network, and hidden Markov model. The widely used emotion recognition research methods mainly include statistical emotion recognition methods, mixed emotion recognition methods, and emotion recognition methods based on knowledge technology (Liu et al., 2017).

#### Statistical Emotion Recognition Method

Statistical emotion recognition methods generally use different types of supervised machines to learn algorithms. The system first inputs the marked information into the algorithm for the system to learn and predict the appropriate emotion category. The process of statistical identification generally has two sets of data, one is a training data set and the other is a test data set. The training data set is used to learn the attributes of the information data, and the test data set is used to evaluate the performance of the supervised machine learning algorithm. Different from other research methods, the accuracy of machine learning algorithms in classification is often relatively high. At the same time, this requires that the machine must have a powerful training data set to support the training of the model in the process of data classification. Common machine learning algorithms include support vector machines, artificial neural networks, and long short-term memory networks.

**(1) Support vector machine**

**FIGURE 2:**
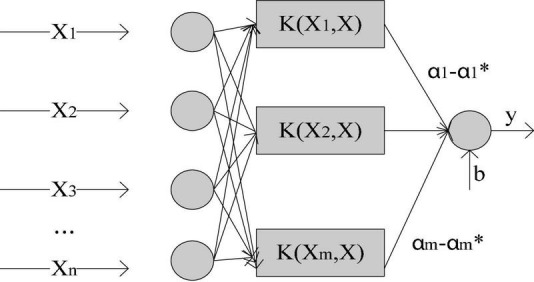
Support vector machine model structure.

The full name of Support Vector Machine (SVM) is Support Vector Machine, and the model structure is shown in Figure 2:

It aims to find a hyperplane to segment data information. The principle of processing is to maximize the data interval, where the interval includes function interval and geometric interval. Assume that for a two-dimensional linearly separable space, data set s = {(x^(i)^,y^(i)^);(i = 1,2,⋯,m)}, of which y ∈ {1,−1}. Define the hyperplane equation as: w^T^x + b = 0, where w is the hyperplane normal vector. For sample x^(i)^ function interval, γ^(i)^ = y^(i)^(w^T^x + b). At the same time, when parameter w,b is scaled, the function interval will be scaled accordingly, so w is constrained to make ||w|| = 1, at this time, the function interval is the geometric interval. When w^T^x + b > 0, the sample label is y = 1; when it is w^T^x + b < 0, the sample label is y = −1. Therefore, the distance between the hyperplane and the two types of data should be as far as possible, that is, the idea of SVM is: first seek the shortest geometric interval on the data set and then maximize the geometric interval. So the goal can be written as (Hofmann et al., 2021):


(1) $$\text{max}\,\frac{2}{||\text{w}||}\,\text{s}.\text{t}.{\text{y}}^{\left(\text{i}\right)}\,\left({\text{w}}^{\text{T}}\,{\text{x}}^{\left(\text{i}\right)}+\text{b}\right)\gg 1,\text{i}=1,2,\cdots ,\text{m}$$

In order to facilitate the calculation, formula 1 is transformed into formula 2:


(2) $$\text{min}\,\frac{1}{2}\,{||\text{w}||}^{2}\,\text{s}.\text{t}.{\text{y}}^{\left(\text{i}\right)}\,\left({\text{w}}^{\text{T}}\,{\text{x}}^{\left(\text{i}\right)}+\text{b}\right)\gg 1,\text{i}=1,2,\cdots ,\text{m}$$

The above formula is a convex quadratic optimization problem, and the Lagrange multiplier method is used to find the optimal solution. Then the Lagrangian function is expressed as (Aqlan, 2020):


(3) $$\text{L}\,\left(\text{w},\text{bα}\right)=\frac{1}{2}\,{||\text{w}||}^{2}+\sum_{\text{i}=1}^{\text{m}}\alpha_{\text{i}}\,\left({\text{y}}^{\left(\text{i}\right)}\,\left({\text{w}}^{\text{T}}\,{\text{x}}^{\left(\text{i}\right)}+\text{b}\right)-1\right),\text{i}=1,2,\cdots ,\text{m}$$

According to Lagrangian duality, the dual problems of all primitive problems are expressed as minimax problems:


(4) $$\max_{\text{α}}\text{​}\min_{\text{w,b}}\text{​}(\text{w,b,α})$$

First obtain the partial derivative of w,b to get (Liu et al., 2018b):


(5) $$\frac{\partial \text{L}}{\partial \text{w}}=\text{w}-\sum_{\text{i}=1}^{\text{m}}\alpha_{\text{i}}\,{\text{y}}_{\text{i}}\,{\text{x}}_{\text{i}}$$

(6) $$\frac{\partial \text{L}}{\partial \text{b}}=\sum_{\text{i}=1}^{\text{m}}\alpha_{\text{i}}\,{\text{y}}_{\text{i}}$$

Let formula 5 and formula 6 be 0, it can get formula 7 and formula 8:


(7) $$\text{w}=\sum_{\text{i}=1}^{\text{m}}\alpha_{\text{i}}\,{\text{y}}_{\text{i}}\,{\text{x}}_{\text{i}}$$

(8) $$\sum_{\text{i}=1}^{\text{m}}{\text{a}}_{\text{i}}\,{\text{y}}_{\text{i}}=0$$

Substituting formula 7 and formula 8 into the Lagrangian function, we get:


(9) $$\min_{\text{w},\text{b}}\left(\text{w},\text{b},\alpha \right)=\sum_{\text{i}=1}^{\text{m}}\alpha_{\text{i}}-\frac{1}{2}\,\sum_{\text{i}=1}^{\text{m}}\sum_{\text{j}=1}^{\text{m}}{\text{y}}^{\left(\text{i}\right)}\,{\text{y}}^{\left(\text{j}\right)}\,\alpha_{\text{i}}\,\alpha_{\text{j}}\,\left\langle {\text{x}}^{\left(\text{i}\right)},{\text{x}}^{\left(\text{j}\right)}\right\rangle$$

(10) $$\text{s}.\text{t}.\alpha_{\text{i}}\gg 0,\text{i}=1,2,\cdots ,\text{m}$$

(11) $$\sum_{\text{i}=1}^{\text{m}}\alpha_{\text{i}}\,{\text{y}}^{\left(\text{i}\right)}=0$$

After the solution is solved, it can be calculated according to formula 5 and formula 6, and then calculated, and finally the hyperplane formula (Huang et al., 2019):


(12) $$\text{f}\,\left(\text{x}\right)={\text{w}}^{\text{T}}\,\text{x}+\text{b}=\sum_{\text{i}=1}^{\text{m}}\alpha_{\text{i}}\,{\text{y}}^{\left(\text{i}\right)}\,{\text{x}}_{\text{i}}^{\text{T}}\,\text{x}+\text{b}$$

The above method can be used for low-dimensional linearly separable cases. For the case of non-linearity, it is necessary to use the kernel function to map the low-dimensional space to the high-dimensional space for processing. This is also the meaning of the introduction of dual expressions in the above derivation. For the solution, the sequence minimum optimal algorithm is usually used.

**(2) Artificial neural network**

**FIGURE 3:**
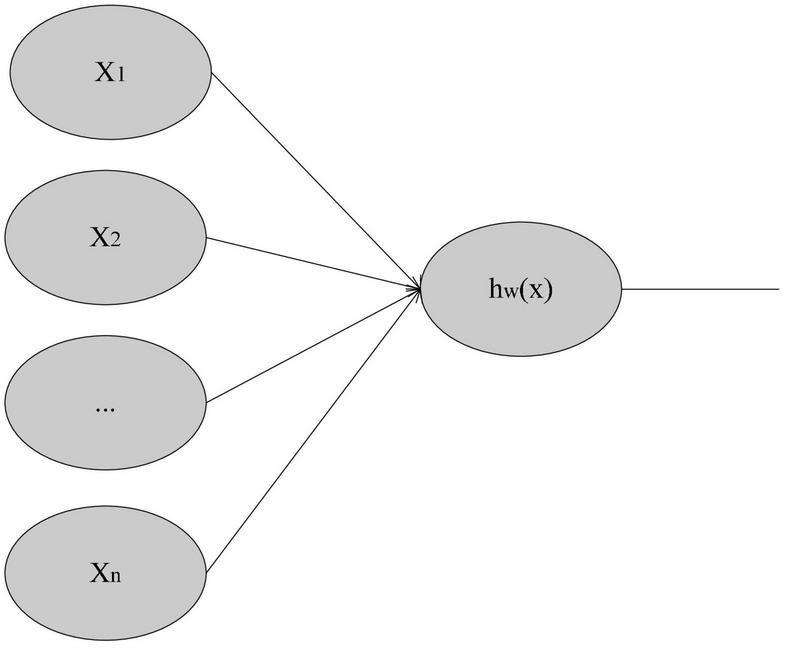
Neuron model.

In 1943, the neuron model was first proposed, as shown in Figure 3:

The input x comes from the training sample (x^(i)^,y^(i)^), and the non-linear function h_w_(x) can be fitted to y. Among them, the non-linear function h_w_(x) has trainable parameters w and b, of which b can be ignored (Wei and Zhao, 2019).

**FIGURE 4:**
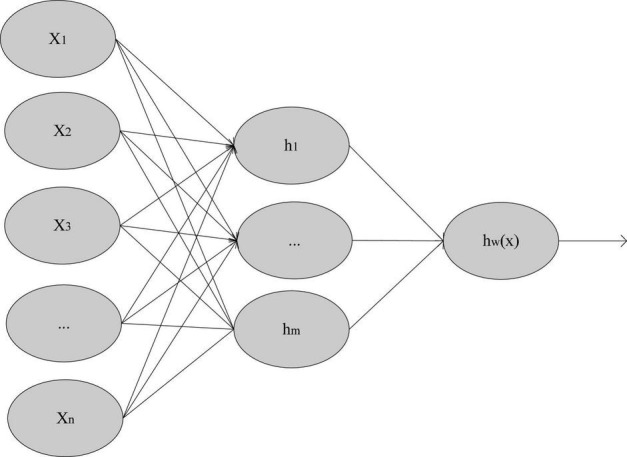
Neural network model.

Artificial Neural Network (ANN) is called Artificial Neural Network. As shown in Figure 4, it has very powerful non-linear data fitting ability.

For the neural network model in Figure 3, suppose that the number of network layers is n_1_, and the network parameters are W = (W^(1)^,W^(2)^,⋯,W^(nl−1)^). Where ${\text{W}}_{\text{ij}}^{\text{l}}$ represents the connection parameter between the first neuron of the l-th layer network and the i-th neuron of the l + 1th layer network. ${\text{h}}_{\text{i}}^{\text{l}}$ represents the activation value of the i-th neuron in the l-th network. For the backpropagation algorithm, assuming the training sample {(x^(1)^,y^(1)^),(x^(2)^,y^(2)^),⋯,(x^(m)^,y^(m)^)}, the classification label is two classification labels, then the sample cost function is shown in formula 13:


(13) $$\text{J}\,\left(\text{W};\text{x},\text{y}\right)=-\text{y}\,\log\left({\text{h}}_{\text{w}}\,\left(\text{x}\right)\right)-\left(1-\text{y}\right)\,\log\left(1-{\text{h}}_{\text{w}}\,\left(\text{x}\right)\right)$$

After adding regularization, the overall cost function of the sample set is (Bakhshi et al., 2020):


(14) $$\text{J}\,\left(\text{W}\right)=\frac{1}{\text{m}}\,\sum_{\text{i}=1}^{\text{m}}\text{J}\,\left(\text{W};{\text{x}}^{\left(\text{i}\right)},{\text{y}}^{\left(\text{i}\right)}\right)+\lambda \,\sum_{\text{j}=1}^{{\text{n}}_{\text{l}}}{||{\text{W}}^{\left(\text{j}\right)}||}_{2}$$

Update the network parameters through the gradient descent method, as shown in formula15:


(15) $${\text{w}}_{\text{ij}}^{\left(\text{l}\right)}={\text{w}}_{\text{ij}}^{\left(\text{l}\right)}-\alpha \,\frac{\partial }{\partial {\text{w}}_{\text{ij}}^{\left(\text{l}\right)}}\,\text{J}\,\left(\text{W}\right)$$

Where α is the learning rate.

**(3) Long and short-term memory network**

The long short-term memory network (LSTM) is called Long Short-Term Memory, it is mainly used to solve the problem of gradient disappearance when long-distance dependence. In order to solve the problem that other algorithms are more sensitive to the input state with a short time step, LSTM adds another state, called the cell unit state, which is used to save the input state with a long time step. For long-term state control, LSTM uses three gates as control switches, which are forget gate, input gate and output gate. First of all, the forgetting gate determines how much of the unit state c_t−1_ at the previous moment can be retained to the unit state c_t_ at the current moment, so that the information that is long before the time step can be saved. It can be expressed by formula 16 (Revathi et al., 2018):


(16) $${\text{f}}_{\text{t}}=\sigma \,\left({\text{W}}_{\text{f}}\cdot \left[{\text{h}}_{\text{t}-1},{\text{x}}_{\text{t}}\right]+{\text{b}}_{\text{f}}\right)$$

Among them, W_f_ is the weight matrix of the forget gate, [h_t−1_,x_t_] is the growth vector of the splicing of these two vectors, and σ is the sigmod activation function. For the input gate, it is to determine how much of the input x_t_ of the current network can be retained to the unit state c_t_ at the current moment, using the formula:


(17) $${\text{i}}_{\text{t}}=\sigma \,\left({\text{W}}_{\text{i}}\cdot \left[{\text{h}}_{\text{t}-1},{\text{x}}_{\text{t}}\right]+{\text{b}}_{\text{i}}\right)$$

Among them, W_i_ is the weight matrix of the forget gate, so that the current input state can be updated according to the input of the previous time step and the current input $\tilde{{\text{c}}_{\text{t}}}$ :


(18) $$\tilde{{\text{c}}_{\text{t}}}=\text{tanh}\,\left({\text{W}}_{\text{c}}\cdot \left[{\text{h}}_{\text{t}-1},{\text{x}}_{\text{t}}\right]+{\text{b}}_{\text{c}}\right)$$

Therefore, the current input unit state $\tilde{{\text{c}}_{\text{t}}}$ and the long-term memory c_t−1_ of the previous time step are combined using the forget gate and the input gate to obtain the current unit state c_t_, as shown in formula 19:


(19) $${\text{c}}_{\text{t}}={\text{f}}_{\text{t}}\cdot {\text{c}}_{\text{t}-1}+{\text{i}}_{\text{t}}\cdot \tilde{{\text{c}}_{\text{t}}}$$

The last is the output gate, which is used to control how much of the current unit state c_t_ can be retained to output h_t_. It also controls the impact of long-term memory on the current output, as shown in formula 20:


(20) $${\text{h}}_{\text{t}}={\text{o}}_{\text{t}}\,\text{tanh}\,\left({\text{c}}_{\text{t}}\right)$$

Where o_t_ is the output gate control (Huimin et al., 2017):


(21) $${\text{o}}_{\text{t}}=\sigma \,\left({\text{W}}_{\text{o}}\cdot \left[{\text{h}}_{\text{t}-1},{\text{x}}_{\text{t}}\right]+{\text{b}}_{\text{o}}\right)$$

**FIGURE 5:**
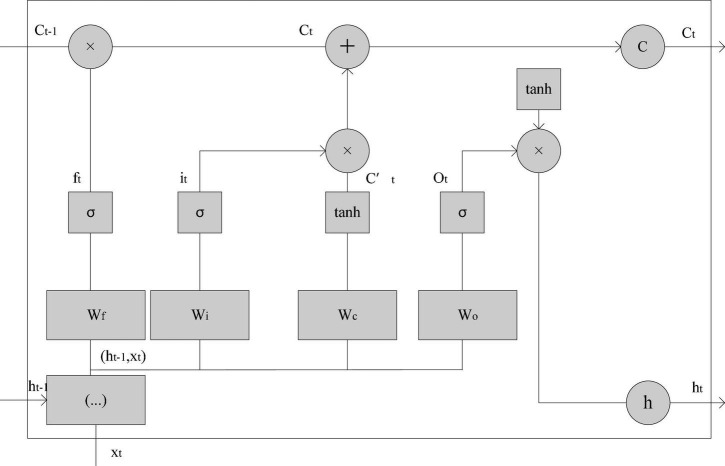
The overall structure of the long and short-term memory network.

So the overall structure is shown in Figure 5 (Demis et al., 2017):

#### Emotion Recognition Method Based on Knowledge Technology

**FIGURE 6:**
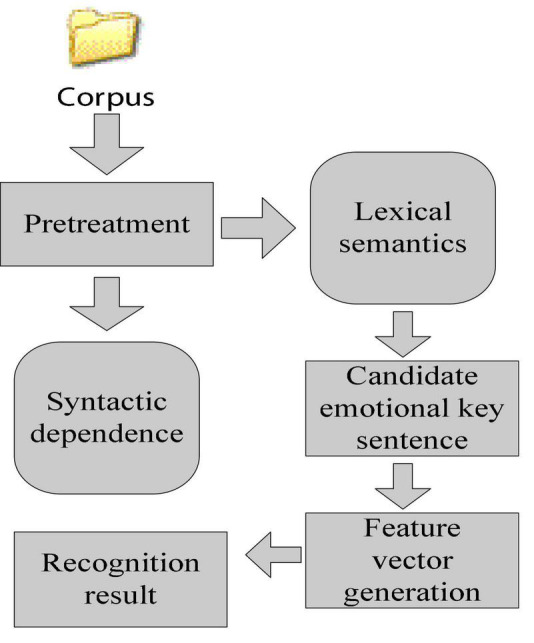
Emotion recognition process based on knowledge technology.

Knowledge technology is actually dictionary technology. The emotion recognition method based on knowledge technology is to recognize emotion by using knowledge or semantic information and features in the emotional field. As shown in Figure 6, the advantage of identifying emotions on the basis of analyzing knowledge is the availability of knowledge resources, which results in the economy and accessibility of information recognition. But it also has its own limitations, that is, the understanding of knowledge or semantics is usually complicated. The existing machine learning algorithm technology cannot accurately understand the small differences between different knowledge and semantics. The realization of knowledge technology mainly includes dictionary and corpus (Wei et al., 2018; Yong et al., 2020; Woo et al., 2021). The dictionary approach is that the system searches for knowledge keywords or opinion information in the dictionary, and at the same time retrieves the corresponding synonyms and antonyms, so as to derive the initial opinion content and sentiment list. The corpus approach is to use knowledge keywords or opinion information as a starting point to search for other phrases with similar characteristics in the context to derive the database from a massive corpus (Rongpeng et al., 2017; Jing et al., 2021). The difference between a corpus and a dictionary is that the corpus takes into account the context of the text environment.

#### Mixed Emotion Recognition Method

The mixed emotion recognition method is actually the product of the combination of the statistical emotion recognition method and the knowledge-based emotion recognition method. It is the complement of the two methods (Yishu and Guifang, 2021; Zheng et al., 2022). It combines the advantages of the statistical emotion recognition method with the advantages of the knowledge-based emotion recognition method. Compared with the other two research methods, the mixed emotion recognition method has the best classification effect. But at the same time, its computational complexity is also the highest, and the requirements for system performance are also high (Liu et al., 2018a; Paweł et al., 2020).

## Predictive Model Test Based on Emotion Recognition Algorithm

**FIGURE 7:**
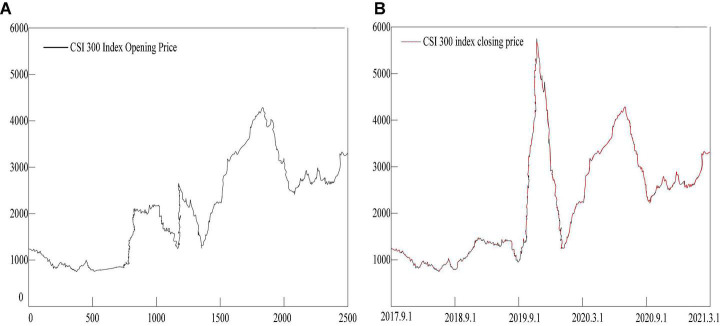
CSI 300 index closing price. **(A)** Shows the opening price of the Shanghai and Shenzhen 300 Index. **(B)** Shows the closing price of the Shanghai and Shenzhen 300 Index.

In this paper, three algorithm models of support vector machine (SVM), artificial neural network (ANN), and long and short-term memory network (LSTM) in the statistical emotion recognition method are selected to predict the operation of the financial market. It uses the daily prices of the Shanghai and Shenzhen 300 Index from September 2017 to March 2021 as the experimental sample data set for model training and learning, as shown in Figure 7. The CSI 300 Index is the most representative index of major constituent stocks in the financial market, reflecting the general direction of the financial market and economic development. The reason why the large-cap index is selected as the sample experimental data is that the large-cap is a concentrated expression of the overall financial market volatility, which can rule out the contingency of violent fluctuations in individual stocks.

The statistical description results of the daily price of the Shanghai and Shenzhen 300 Index from September 2017 to March 2021 are shown in Table 1:

**TABLE 1:** Statistical description of the closing price.

CSI 300 index	All samples	Test sample
Sample size	3,000	120
Mean	2165.126	3512.67
Standard deviation	1035.175	91.477
Covariance	1143714	8234.156
Maximum	4933.158	3607.579
Minimum	693.648	3144.349
Skewness	0.4215	0.5188
Kurtosis	2.6174	2.6403

It can be seen from Table 1 that the skewness values of the opening and closing prices of the Shanghai and Shenzhen 300 Index are positive, and the kurtosis values of all its samples are greater than 2. This shows that the opening and closing price indices do not follow a normal distribution.

The main structural parameters of the prediction model of the emotion recognition algorithm are shown in Table 2:

**TABLE 2:** The main structural parameters of the prediction model.

Algorithm model	SVM	ANN	LSTM
Input dimension	44	29	16
Number of hidden layer neurons	18	10	6
Hidden layer transfer function	Sigmoid tangent	Sigmoid tangent	Sigmoid tangent
Input layer transfer function	Pure-linear transfer function	Pure-linear transfer function	Pure-linear transfer function
Training function	Levenberg-Marquardt training algorithm	Levenberg-Marquardt training algorithm	Levenberg-Marquardt training algorithm

### Support Vector Machine (SVM) Model Testing

**FIGURE 8:**
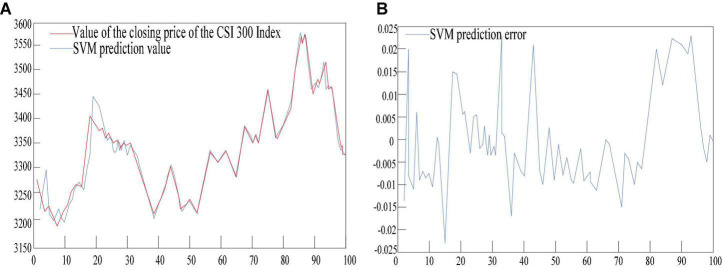
Support vector machine model testing. **(A)** Is a simulation diagram of the test results of the support vector machine model. **(B)** Shows the error of the test results of the support vector machine model.

It can be seen from Figure 8 and Table 3 that the number of index variables of the support vector machine model is 50. The average absolute error of the test sample fitting is 24.343, the mean square error is 912.81, and the root mean square error is 34.175. The average absolute error percentage is 0.0069, the mean square error percentage is 0.0000854, and the root mean square absolute error percentage is 0.0089. The average absolute error ratio is 06915, the mean square absolute error ratio is 0.0086, and the root mean square absolute error ratio is 0.0917.

**TABLE 3:** SVM model performance evaluation form.

Performance	Test sample
Mean absolute error	24.343
Mean square error	912.81
Root mean square error	34.175
Mean absolute error percentage	0.0069
Mean square error percentage	8.54E-05
Root mean square error percentage	0.0089
Mean absolute error ratio	0.6915
Mean square absolute error ratio	0.0086
Root mean square absolute error ratio	0.0917
Number of indicators	50

### Artificial Neural Network (ANN) Model Test

**FIGURE 9:**
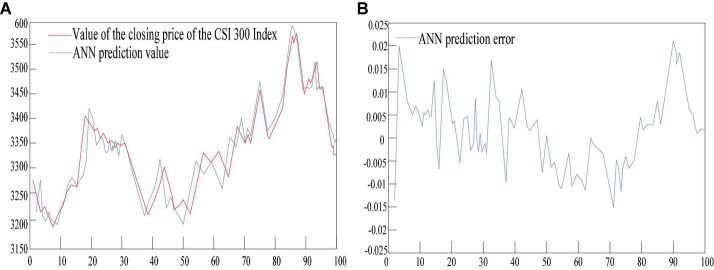
Artificial neural network model test. **(A)** Is a simulation diagram of artificial neural network model test results. **(B)** Shows the error of the artificial neural network model test result.

The performance evaluation results of the artificial neural network model are shown in Table 4. It can be seen from Figure 9 and Table 4 that the number of index variables of the artificial neural network model is 50. Among them, the average absolute error of the test sample fitting is 21.674, the mean square error is 906.47, and the root mean square error is 28.796. The average absolute error percentage is 0.0069, the mean square error percentage is 0.000084, and the root mean square absolute error percentage is 0.0079. The average absolute error ratio is 0.6457, the mean square absolute error ratio is 0.0081, and the root mean square absolute error ratio is 0.0937.

**TABLE 4:** ANN model performance evaluation form.

Performance	Test sample
Mean absolute error	22.843
Mean square error	907.15
Root mean square error	29.435
Mean absolute error percentage	0.0071
Mean square error percentage	8.35E-05
Root mean square error percentage	0.0087
Mean absolute error ratio	0.6734
Mean square absolute error ratio	0.0084
Root mean square absolute error ratio	0.0941
Number of indicators	50

## Discussion

Using three models to predict and test the experimental data of the Shanghai and Shenzhen 300 Index, the following conclusions can be drawn:

The experimental data shows that the prediction effect of the support vector machine model is satisfactory in the test samples of closing price data. The gap between the predicted value and the actual value is small, and the prediction accuracy is high. It is feasible to apply it to the stock index trend prediction;

The performance index results show that the artificial neural network model is a more effective model to solve the problem of dimensionality reduction, has good self-adaptation and self-learning capabilities, and can better deal with non-linear problems;

Under the construction of experimental parameters that keep the support vector machine model and the artificial neural network model consistent, the long-term and short-term memory network model is more effective than the other two models in improving the effectiveness of the input variables. However, in the prediction accuracy of the test samples, the performance of the long and short-term memory network model is not as good as the other two models. Generally speaking, in terms of short-term prediction of the market trend, it is practical to use the long and short-term memory network algorithm model to input the data variables of the sample. It supports the prediction application and development of emotion recognition algorithms in financial markets and economic growth and development trends.

The experimental data of the entire model shows that the support vector machine, artificial neural network and long-short-term memory network algorithm models in the emotion recognition algorithm are feasible and effective for the prediction of financial development and economic growth and development trends.

## Conclusion

Financial development, economic growth and development trends are extremely complex and non-linear dynamic processes that are difficult to predict. Because there are many variables that are extremely complex and cannot be calculated effectively in the financial market, each variable has its own characteristics and special purpose, including the limitations brought by the variable itself. So far there is no suitable selection criteria for variables. But what needs to be ensured is that the input variables of the market forecasting model must not only reach the minimum quantity limit, but also must be able to fully reflect market issues. However, selecting too many variables will inevitably make the calculation of data processing by the prediction model more complicated, which invisibly increases the performance requirements of the model algorithm. In addition to the impact of objective factors, some subjective factors can also lead to a decline in forecasting performance, especially some variables that have nothing to do with market trends. This will reduce the accuracy of the prediction and cause undesirable results. The emotion recognition algorithm model can effectively solve these problems. The experimental data in this article also shows that although the three emotion recognition algorithms have different characteristics, they all have their own advantages in the prediction process. This can effectively process and analyze complex information data, and perform high-level simulation and fitting on it. Coupled with the powerful learning ability of the algorithm itself, it has good operability and feasibility in the analysis and forecast of financial development, economic growth and development trend.

Although this article uses emotion recognition algorithms to conduct in-depth research on the status and development trend of financial development and economic growth, there are still many shortcomings. The depth and breadth of the research in this article are not enough. In the course of this research, the selection and acquisition of experimental data were carried out under absolutely ideal conditions. The completeness and effectiveness are not enough, and some interference factors mixed with financial market data are not taken into consideration. The depth of market information tracking and mining is also restricted by many factors. The academic level research is also limited, and the research on financial development, economic growth and development trends is still in the preliminary stage. In future research work, we will analyze the characteristics of the market environment from more angles based on the current technology and level, and continuously optimize algorithms to accurately predict financial development, economic growth and development trends.

## Data Availability Statement

The original contributions presented in the study are included in the article/supplementary material, further inquiries can be directed to the corresponding author.

## Author Contributions

DW: writing. BL: editing. XY: data analysis. All authors contributed to the article and approved the submitted version.

## Conflict of Interest

The authors declare that the research was conducted in the absence of any commercial or financial relationships that could be construed as a potential conflict of interest.

## Publisher’s Note

All claims expressed in this article are solely those of the authors and do not necessarily represent those of their affiliated organizations, or those of the publisher, the editors and the reviewers. Any product that may be evaluated in this article, or claim that may be made by its manufacturer, is not guaranteed or endorsed by the publisher.
